# Perspectives on the utility and interest in a point-of-care urine tenofovir test for adherence to HIV pre-exposure prophylaxis and antiretroviral therapy: an exploratory qualitative assessment among U.S. clients and providers

**DOI:** 10.1186/s12981-020-00308-w

**Published:** 2020-08-06

**Authors:** Ashley R. Bardon, Jane M. Simoni, Leif M. Layman, Joanne D. Stekler, Paul K. Drain

**Affiliations:** 1 Department of Epidemiology, University of Washington, HMC # 359927, 325 9th Avenue, Seattle, WA 98104-2499 USA; 2 Department of Global Health, University of Washington, Seattle, WA USA; 3 Department of Psychology, University of Washington, Seattle, WA USA; 4 Department of Gender, Women, and Sexuality Studies, University of Washington, Seattle, WA USA; 5 Division of Allergy and Infectious Disease, Department of Medicine, University of Washington, Seattle, WA USA

**Keywords:** Perceptions, Point-of-care test, Tenofovir, Adherence, Antiretroviral therapy, Pre-exposure prophylaxis

## Abstract

**Background:**

Real-time, objective measures of adherence to antiretroviral therapy (ART) and pre-exposure prophylaxis (PrEP) are needed to better assess adherence levels and to expedite clinical response for those with suboptimal adherence. Point-of-care tenofovir (POC-TFV) testing has been proposed as a solution to facilitate real-time antiretroviral adherence monitoring, but little is known about how health care providers, people living with HIV (PLWH) receiving ART, and people receiving PrEP will perceive POC-TFV testing.

**Methods:**

We conducted an exploratory qualitative study to assess perspectives on the utility and interest in POC-TFV testing from potential end users. We conducted three focus group discussions (FGDs) among 17 PLWH receiving ART and four individuals receiving PrEP, as well as eight in-depth interviews (IDIs) with health care providers in the Seattle area and presented participants with a hypothetical urine-based POC-TFV test. FGDs and IDIs were audio recorded, transcribed, coded, and analyzed to describe emerging themes.

**Results:**

Overall, study participants demonstrated divergent opinions about the POC-TFV test. Among study participants, PLWH were most ambivalent about POC-TFV testing, first demonstrating reluctance to TFV-level monitoring and shifting positions during the FGDs. However, all PLWH participants were receptive to POC-TFV testing if requested by their provider. PrEP participants were generally supportive of POC-TFV testing for routine adherence monitoring and emphasized potential value in self-administered testing. Providers’ perceptions were equally divided – half suggested POC-TFV testing would be valuable, particularly for people receiving PrEP, while half indicated the test would have little benefit for most individuals receiving ART or PrEP in the U.S. All providers agreed that POC-TFV test results could be beneficial for assessing discrepancies in viral load results and self-reported adherence among PLWH. The study also revealed that a low-cost, non-urine-based POC-TFV test with a long-term limit of detection would be preferred over the hypothetical urine-based test.

**Conclusions:**

Our findings indicate POC-TFV testing may be beneficial for routine, clinic-based adherence monitoring, particularly for individuals receiving PrEP or for PLWH with persistent viremia or following recent ART initiation. These findings should also be used to formulate a target product profile for a POC-TFV test and to guide further developments in tools for objective antiretroviral adherence monitoring.

## Background

Approximately 22 million people living with HIV (PLWH) are receiving antiretroviral therapy (ART), [1] and an estimated 380,000 people in 68 countries are receiving oral pre-exposure prophylaxis (PrEP) for the prevention of HIV [2]. Lifelong adherence to ART is necessary for PLWH to avoid poor health outcomes and reduce the risk of onward HIV transmission [3]. Similarly, PrEP is only effective in preventing HIV acquisition for those who maintain optimal levels of adherence [4, 5]. Therefore, assisting individuals in maintaining adherence to ART or PrEP may help reduce HIV transmission and prevent the emergence of drug-resistant HIV [3, 6–11].

A common practice of assessing ART adherence is by testing HIV RNA viral load (VL) [1], but a VL test result is not consistently analogous to recent ART adherence [10, 12, 13]. In addition, subjective methods used to measure ART and PrEP adherence, such as self-reported adherence, are prone to social desirability bias; while commonly used objective measures, such as pill counts and pharmacy refill records, are often inaccurate and unreliable [14–16]. Objective metrics are available to measure adherence through pharmacokinetic analysis of concentrations of tenofovir (TFV), a drug formulation found in oral PrEP regimens and most ART regimens, which is detectable in whole blood, plasma, dried blood spots, cerebrospinal fluid, mucosal fluid, oral tissue, urine, and hair [12, 17–30]. However, widespread implementation of these metrics is limited by its expense, turnaround time, and need for specialized personnel [23, 25]. Thus, more scalable, timely, and cost-effective methods for adherence monitoring are needed to provide an objective assessment of ART and PrEP adherence [15, 23, 25].

Real-time measures of TFV may facilitate identification of PLWH or PrEP clients with adherence challenges before adverse clinical outcomes occur [12, 14, 24, 25, 27, 28, 31–34]. Adherence testing has also proven to be more reliable in assessing medication adherence than self-report or electronic monitoring tools [12, 14]. Several tests that measure levels of TFV in real time at the point of care are currently in development [24, 25, 28, 35], and may impact clinical care and patient outcomes. Recent studies have found TFV testing to be acceptable among PrEP participants and beneficial in improving accuracy of self-reported adherence, motivating better medication adherence, and enhancing adherence counseling and support [23, 27, 32, 36]. However, little is known about how providers and PLWH receiving ART would perceive point-of-care (POC) TFV testing and how PrEP clients would perceive TFV testing in real time. We sought to expand on previous findings and assess clients’ and providers’ perspectives on POC-TFV testing, specifically the perceived utility and interest in testing, through an exploratory qualitative analysis.

## Methods

### Study design

Between February and April 2019, we conducted three focus group discussions (FGDs) among adult PLWH receiving ART and PrEP clients at a large, Ryan White-funded HIV clinic that also provides PrEP care in Seattle, Washington. We also conducted eight semi-structured in-depth interviews (IDIs) among providers of HIV and PrEP medical care in the greater Seattle area.

### Study population

We conducted two separate FGDs among PLWH receiving ART. The first FGD included individuals with a suppressed VL (< 200 copies/mL) in the prior six months; the second FGD included those with an unsuppressed VL (≥ 200 copies/mL) in the prior six months. We conducted a third FGD with PrEP clients. Individuals were eligible to participate if they were 18 years or older, spoke English, were willing and able to provide informed consent, and, for PLWH, had been on a treatment regimen containing tenofovir disoproxil fumarate or tenofovir alafenamide for at least six months. All individuals who met eligibility criteria were identified by an electronic medical record query completed by the University of Washington Center for AIDS Research (CFAR). Eligible individuals were then selected for recruitment via convenience sampling and were recruited directly during routine clinic visits or by phone by the CFAR HIV research coordinator. In total, 45 individuals agreed to participate, and 21 individuals presented for the FGDs and provided oral consent to participate.

We conducted eight IDIs among medical providers who were currently providing medical care for PLWH or PrEP clients. Providers were eligible to participate if they were able to prescribe medications, spoke English, and were willing and able to provide informed consent. Eligible providers were purposively identified by one of the study investigators (JDS) to intentionally achieve a diverse sample of providers in terms of gender, clinic type and location, and number of years providing care for ART and/or PrEP clients. Nineteen providers were recruited via email by JDS and ARB, and one provider voluntarily recruited a colleague to participate in the study. Of the 20 providers contacted, eight responded and agreed to participate.

### Study procedures and data collection

The FGDs were conducted in a conference room in the HIV clinic from which participants were recruited. Participants completed a self-administered, paper-based demographics questionnaire prior to the FGD and were each provided with a meal, parking validation, and a USD $35 gift card as compensation for their time and travel expenses. The FGDs lasted a mean of 53 min (range: 43–68 min). JMS facilitated each discussion; ARB took detailed notes; and LML, JDS, and PKD were also present during the FGDs. Participants were not acquainted with any members of the research team prior to the FGDs.

We conducted and digitally recorded eight IDIs with providers using HIPAA-compliant teleconference software. IDI participants were not offered compensation for their time. Each participant completed a short demographics questionnaire prior to the interview. IDIs were facilitated by ARB using a semi-structured interview guide and lasted a mean of 24 min (range: 17–31 min). No other members of the research team were present during the interviews.

Questions and discussion topics for the FGD and IDI guides were developed a priori based on a literature review and consultations with clinical and behavioral experts. The guides probed participants’ views on current adherence monitoring practices, possible utilities of the POC-TFV test, motivations for its use, target populations for the test, potential settings for conducting the test, feasibility of integrating POC-TFV testing in a clinical care setting, and anticipated effects of TFV testing. All participants were presented with characteristics of a POC-TFV test based on an assay that has been developed and validated [24, 25, 28]. Prior to beginning each FGD and IDI, we described a hypothetical POC-TFV test to participants. The test was described as a simple, qualitative lateral flow assay (LFA) that measures recent (prior two to three days) TFV concentrates in urine in real time. FGD participants were visually presented with an over-the-counter pregnancy test as an example of a urine-based qualitative LFA. Specific details about the purpose of using the test, how and where it would be conducted, and its validity and reliability were not provided to participants to allow for an open discussion about potential use cases for the test.

### Data analysis

We developed one codebook for FGDs and another for IDIs; these were deductively built on domains outlined in the discussion and interview guides and inductively expanded to include additional domains introduced by participants. The final version of the codebook was agreed upon by ARB, JMS, and LML. All FGDs and IDIs were transcribed by an external transcription service and were checked for accuracy by ARB. ARB and LML independently coded each transcript, exchanged transcripts to ensure consistency, and discussed discrepancies and re-coded as necessary. After reaching consensus for all coded transcripts, we performed qualitative queries to identify emergent themes and compared findings between groups. All data were managed, coded, and analyzed using Dedoose v8.2 software.

### Ethics

All participants provided oral consent prior to participation, and FGD participants used fictitious names to ensure anonymity. The study was approved by the Institutional Review Board at the University of Washington.

## Results

Demographic characteristics for FGD and IDI participants are described in Table 1. Most PLWH had been receiving ART for more than 10 years (69%), and most PrEP participants had been receiving PrEP for at least one year (75%). Among the eight IDI participants, seven had a Doctor of Medicine degree, and one had a Nurse Practitioner degree. IDI participants provided care in a private practice setting (n = 2), non-profit health center (n = 2), academic health center (n = 3), or non-profit hospital (n = 1). Seven of the IDI participants provided care for both PLWH and PrEP clients, and one provided care for only PrEP clients. Several themes emerged from the FGDs and IDIs, which are described in detail below.

**Table 1:** Demographics of focus group discussion and in-depth interview participants (N = 28)

	HIV-positive individuals on ART		HIV-negative individuals on PrEP (n = 4)	Providers (n = 8)
	Viral load < 200 copies/mL (n = 10)*	Viral load ≥ 200 copies/mL (n = 6)**		
Gender				
Male	6	5	4	4
Female	3	1	0	4
Transgender female	1	0	0	0
Age, median (range)	57 (39–65)	48 (32–57)	35 (31–55)	49 (38–65)
Race				
American Indian/Alaskan Native	2	0	0	0
Asian	1	0	0	3
Black/African American	4	3	0	0
Native Hawaiian/Pacific Islander	0	1	0	0
White	3	2	3	5
Other	0	0	1	0
Latino/Hispanic/Chicano	0	0	2	0
Highest Level of Education				
Some secondary education/high school (9–12 years)	2	0	0	0
High school graduate or GED	3	5	1	0
College or associates degree	4	1	2	0
Advanced degree	0	0	1	8
Years on HIV medications				
< 1 year	0	1	1	–
1–5 years	1	1	3	–
> 5–10 years	2	0	0	–
> 10 years	7	4	0	–
Years providing care for ART/PrEP				
< 5 years	–	–	–	1
5–20 years	–	–	–	3
> 20 years	–	–	–	4

*GED* general education diploma, *ART* antiretroviral therapy, *PrEP* pre-exposure prophylaxis

*Data missing for one participant for highest level of education

**Data missing for one participant for all characteristics

### Initial reactions and perspectives on POC-TFV testing

Initial reactions and feedback on the POC-TFV test varied significantly among participants. Opinions from PLWH receiving ART were mostly unfavorable when first presented with the concept of the POC-TFV test in the FGDs.

“Personally, I wouldn’t need to do that. Because I know if I’ve taken my meds or not.”–Male PLWH, suppressed VL

However, several PLWH shifted positions during the discussions, and all PLWH were generally supportive of POC-TFV testing by the end of the FGDs. Some PLWH had concerns about overall acceptance of POC-TFV testing, as they suggested it may be invasive to clients’ privacy, specifically with regard to autonomy over their own care. Several PLWH said they would perceive adherence monitoring from their provider as an indication of distrust in their self-reported adherence. Though some still had reservations about how it would be beneficial for their own care, all PLWH participating in the FGDs agreed that they have confidence in their providers’ decisions and would consent to adherence monitoring if it were incorporated into routine care. PrEP participants had mostly positive initial reactions about the POC-TFV test and remained enthusiastic throughout the duration of the FGD.

“So, just some more reassurance. I think that’s a really good idea.”–Male PrEP client.

Half of providers had favorable initial reactions of POC-TFV testing and perceived the test as beneficial in supporting clinical decision-making for their ART and PrEP clients, while the other half found the test to have little or no utility in improving clients’ care and treatment. Providers tended to maintain their initial perceptions throughout the entirety of the interview. Similar to concerns introduced by PLWH, providers also explained how the POC-TFV test could impact the client-provider relationship.

“It seems a little too big sibling-ish to be overseeing every minute thing they do, but I’m actually not too sure yet.”–Male provider, non-profit health center

### Concerns about POC-TFV testing

Several concerns about potential implementation difficulties or negative effects of POC-TFV testing emerged. Some PLWH were not enthusiastic about the test due to fears and privacy concerns about how test results could be used. One PLWH raised concerns about insurance companies obtaining and using adherence data:

“Is this for measuring a person’s pre-existing conditions to insure them in the future? Like they’re given the chance to adhere to their medical treatment, and then they refuse to? And then they’re taking that risk into their own hands, so the insurance company can refuse them?”–Male PLWH, suppressed VL

PLWH participants in the unsuppressed VL group also raised concerns about maintaining confidentiality of their HIV status if they were to use POC-TFV tests at home.

“So, I would not even [bring that home] at all. It’s unsafe for anyone to know – and you don’t want people to know your business if you have it or not.”–Male PLWH, unsuppressed VL

Though the POC-TFV test’s validity was not described to participants, PrEP participants suggested false positive or negative test results may cause some apprehension among end users.

“So what if you get a positive reading? That’s the only other thing is that not all of these will have….Just like a normal pregnancy test, you can always have false positives or false negatives. So that’s the only other thing you have to worry about.”–Male PrEP client

Providers had similar concerns about the test’s validity and the potential for falsely accusing clients of nonadherence, which they perceived as possibly harmful to the client-provider relationship.

Many providers also saw potential difficulties in POC-TFV testing for monitoring PrEP adherence, as they presume some clients are using intermittent or pericoital dosing (i.e., on-demand PrEP) as opposed to the recommended daily dosing.

“I do, again, have the hesitation that, especially for men who have sex with men, which is the majority of individuals on PrEP in our practice, there is fairly good data that four pills a week is probably enough. And the guidelines don’t all agree about pericoital dosing, but a lot of men do know about it. So, I guess I do worry….that if I had a urine test that said they hadn’t taken it in three days, they may still be taking enough to be effective. And while it’s controversial, there are providers in the community prescribing pericoital dosing.”–Male provider, academic health center

### Motivations for POC-TFV testing

Several motivations for POC-TFV testing emerged across all the groups. From PLWH and PrEP participant perspectives, a clinic-based POC-TFV test would be primarily used to monitor adherence to better inform clinical decision making and support for poor adherence. A couple of PLWH participants further clarified that routine adherence monitoring could help providers identify cases of drug resistance and prompt regimen switches earlier.

Another primary use for the POC-TFV test posed by PrEP participants was self-administered testing during a potential exposure event, such as a sexual encounter, as reassurance of their protection from HIV acquisition.

“I think it’s wonderful… I see it more for self-testing than for testing others… That doesn’t seem like it can work. It’s like handing out an HIV test at home and saying, ‘Oh, you wanna have sex? Let me test you.’ Maybe some people would accept it, but I don’t see it realistically working… I think it’s great for monitoring yourself. I think it’s more important that what you can do for yourself instead of thinking of what [someone else] is going to do for you… [I]f another person is not taking care, it’s not your obligation to take care of the other person. You take care of yourself, …that’s what counts to me at this point… So, I think this one, it will be good to make sure your level of protection.”–Male PrEP client

From providers’ perspectives, the most important motivation for POC-TFV testing would be to provide objective evidence as to why certain PLWH are unable to achieve a suppressed VL despite consistently reporting high ART adherence. Some providers shared similar views on utilizing POC-TFV test results to detect risk of drug resistance for those with persistent viremia to help inform regimen changes.

“I think it would – if costs were not an issue – I think it could help give us ideas about who might be at higher risk for developing resistance, especially if they were on a lower-barrier regimen.”–Female provider, non-profit health center

One provider also described a circumstance in which POC-TFV testing could be informative in clinical decision-making for clients on TFV-containing regimens with nephrotoxicity:

“Well, I would say in our patients with renal failure, that dosing tenofovir can be really tricky because I think it’s like an every three-day dose or something like that, and I never quite know for achieving the right level. I think in patients with renal failure, in particular, this test would be helpful.”–Female provider, non-profit hospital

Additional uses voiced by all participants in favor of conducting POC-TFV testing were to motivate accuracy in self-reported adherence, improve short-term adherence prior to clinic visits or long-term adherence, and support adherence counseling with individuals receiving ART or PrEP by providing real-time evidence of medication levels.

### Potential settings for POC-TFV testing

When asked by the research team to consider settings for POC-TFV testing, PLWH primarily focused on its use in a clinical setting, integrated with VL testing and administered by a health care provider.

Alternatively, individuals on PrEP generally discussed using the test in the home or as a self-administered test in other non-clinical settings to monitor their own adherence or to test partners.

“I think it’s great. I think it should be available for home use.”–Male PrEP client

Providers described a number of settings where a POC-TFV test could be utilized, although no consensus was reached on the ideal setting for POC-TFV testing. Most providers acknowledged that integrating it with routine care in the clinic would be feasible, although one provider did not consider POC-TFV testing to be feasible as a part of routine care due to the high volume of their client population.

“[W]e probably have 350 or 400 people on PrEP and another… 400 with HIV, that’s a lot of HIV-related lab testing being done. It’s just a large volume… That’s a lot of burden on the lab.”–Male provider, non-profit health center

Providers described how the test could be used in HIV and sexually transmitted disease (STD) clinics, clinical pharmacies, hospital inpatient departments, other health care establishments, bathhouses, or in the home. For HIV/STD clinic implementation, providers concluded that POC-TFV testing could be integrated with routine care and laboratory testing such as VL monitoring and HIV/STD testing. Providers also described novel use case scenarios for implementation within hospital settings:

“Definitely in the hospital setting, we could use it. Often, in those times, we have patients on ART but also multiple other medications, so it might be helpful just for monitoring to make sure that we’re not dealing with drug-drug interaction or that the patient hasn’t vomited it, or… [is] absorbing it okay.”–Female provider, non-profit hospital

“So, there are instances in which a person comes into the hospital, and because it takes a couple days to get a viral load back, we don’t always know about their adherence right away. So, if it were made available in the inpatient hospital setting, I bet it would get used. Maybe not routinely, but I bet any time there’s a question about adherence, I bet it would be used in inpatient setting.”–Male provider, academic health center

### Potential target populations for POC-TFV testing

All FGD and IDI participants agreed that POC-TFV testing may not be suitable for everyone receiving TFV-containing regimens but that certain populations could benefit, specifically PrEP clients, people who have occasional memory lapses, people who are suspected of misreporting or underreporting adherence challenges, PLWH who have discrepancies in their self-reported ART adherence and VL results, and people who have recently initiated treatment.

One PLWH described how POC-TFV testing may be useful for PLWH newly initiating ART:

“It would be good in the beginning parts. I did a study when I first started…and it was a struggle to get that habit down of taking medications… I could see…with new people who are starting medications, to test… So, if you’re trying to start out and stuff, it would be good to have…to test people.”–Male PLWH, unsuppressed VL

Two PLWH receiving ART described how POC-TFV testing may have been useful while experiencing challenging life circumstances, such as stints of binge drinking or housing displacement:

“If I drank a lot. There was times where I would drink so much, and…I would skip [my medications] for a while. And then, just not even really comprehend it. So, I’d think, ‘Did I take my medication? How long has it been? Has it been a week? Has it been a day?’ Probably wasn’t that dramatic, but it might be nice to have [a test] on hand.”–Male PLWH, unsuppressed VL

“[S]ometimes…I was so focused on homeless stuff and everything else that I wasn’t focused on [taking my medication]. There [were] times where I just didn’t want to take medication at all… But, I mean, if they were going to use these, then I would say they would be good for that.”–Male PLWH, unsuppressed VL

Additionally, one provider explained that POC-TFV testing could be beneficial for assessing adherence in individuals with an unknown adherence history, for example those who are new to a clinic:

“I guess the other instance in which it might be useful is for those people, either… newly diagnosed or newly starting ART, or new to my clinic who I really don’t have a long history of labs, I don’t have a long history of rapport, and don’t really know their ability to adhere.”–Male provider, academic health center

Another provider suggested that POC-TFV testing could be useful for adherence monitoring in other patient populations prescribed to TFV, such as individuals with hepatitis B infection:

“I would definitely consider using it. Yeah, I wanted to add: I see a lot of patients with hepatitis B, which of course is not ART, but we do use tenofovir for hepatitis B. In some ways, I have more problems with that population because it’s monotherapy… Sometimes I’m not able to achieve an undetectable viral load, and I’m not sure if it’s because the patient is not telling – not being compliant – and not telling me, if there’s some absorption issues, or what… I could see how this would actually be more helpful in my hepatitis B practice, in some ways, than my HIV practice.”–Female provider, non-profit health center

### Preferences on POC-TFV test attributes

Though participants were not directly queried about test attributes and characteristics, many shared their thoughts on some aspects of the test that would be preferable or that would deter them from using the test. As mentioned in the findings above, many PrEP participants indicated that a home-based test would be preferable to clinic-based TFV testing. Participants did not directly state that cost would be a barrier to either clinic-based or self-administered POC-TFV testing, but when asked by the facilitator if anyone would pay for this type of test for home use, one PrEP participant said:

“$12. I mean, how much does a pregnancy test cost? They are at the pharmacy at Walgreens… I think it should be the same amount.”–Male PrEP client

Regarding the specimen type, some providers had specific concerns about using urine to measure TFV, as they suspect clients may correlate urine-based tests with illicit drug testing:

“I think urine tests sometimes make people feel like they’re being not trusted, whether it’s a U-Tox… I do think that a lot of people could be triggered by giving a urine test.”–Female provider, non-profit health center

Another characteristic that was raised by a provider was the test’s limit of detection, emphasizing that a POC-TFV test that could measure long-term adherence would be preferable to the short-term adherence test described to participants in this study.

“If it was like an [hemoglobin] A1C, and it estimated their tenofovir use over the last three months, I think that might be more valuable.”–Male provider, academic health center

## Discussion

Findings from this exploratory qualitative study provide important insights on clients’ and providers’ perceptions of a new tool that could be integrated into care to monitor recent ART or PrEP adherence levels in real time. Themes identified in this study indicate that providers may benefit from clinic-based POC-TFV testing by gaining an even clearer account of their clients’ health, particularly for some people receiving PrEP and PLWH receiving ART who have discrepancies in their self-reported adherence and VL results. However, participants’ views were divided on the test’s overall utility and value for all people receiving TFV-based regimens. PLWH and PrEP participants perceived clinic-based POC-TFV testing as potentially beneficial for improving care, though PLWH participants had some ambivalence about TFV-level monitoring due to concerns about privacy, confidentiality, and autonomy of their care and treatment. In addition, PrEP participants stated that home-based POC-TFV testing could provide some reassurances regarding their protection from HIV acquisition.

Few studies have assessed clients’ and providers’ interest in TFV testing; therefore, there is limited research with which we can contextualize our findings. However, our findings are consistent with those from previous studies in the level of interest in TFV testing among PrEP participants, perceived applications for TFV testing in clinical settings, and concerns with TFV testing. Other recent studies have found TFV testing to be useful among PrEP participants and may result in more accurate self-reported adherence and more engaged counseling and support for those who experience adherence challenges [23, 27, 32, 36]. Female PrEP participants in sub-Saharan Africa also reported that TFV testing would motivate them to improve their adherence and recommended that real-time TFV monitoring be integrated into standard of care [23]. Another recent study found high acceptability of TFV monitoring among U.S. PrEP participants, strong interest in self-administered testing, and concerns about being tested for illicit drugs and “white coat adherence”, or an increase in adherence prior to a clinic visit [32]. Our study found similar levels of interest from participants and similar concerns about the perception of illicit drug testing. Our study expanded on previous findings from PrEP participants to also include perspectives from PLWH receiving ART and health care providers.

Findings from this study revealed several perceived weaknesses of urine-based POC-TFV testing, which may be informative in guiding development of other objective adherence monitoring tools. First, the test presented to participants in this study had a short-term limit of detection for TFV, which was perceived by most participants as problematic and possibly impractical for drawing conclusions about a clients’ overall adherence. Most participants in the study acknowledged that a longer-term TFV detection limit would be more valuable in assessing medication adherence and guiding clinical care decisions, such as adherence counseling or regimen switches. A lower detection limit may also help to prevent test result biases from “white coat adherence”. This study also revealed the POC-TFV test’s limitations in measuring adherence in some PrEP clients. As one provider reflected in the IDI, POC-TFV testing among PrEP clients who are adopting pericoital, or on-demand, PrEP may not be practical and may lead to false accusations of nonadherence. Although the POC-TFV test’s validity and reliability were not specified in the FGDs and IDIs, the test’s sensitivity and specificity were predominant concerns from many participants, as false positive or negative results may cause undue harm to clients and to the client-provider relationship. Other test attributes that were perceived by study participants as important factors in uptake and scale-up of POC-TFV testing were the cost of the test and the specimen type, with preferences for a low-cost test and a non-urine-based test.

Our study has several limitations that should be considered. Findings from this exploratory study may not be representative of views from other U.S. and non-U.S. clients and providers. ART and PrEP clients were recruited for this study via convenience sampling from a single clinic serving the greater Seattle area, and we also experienced a high no-show rate among clients recruited for the FGDs and a high degree of non-response among providers recruited for the IDIs; therefore, our findings may have been impacted by sampling and selection biases. The study was also limited by its small sample size, particularly among PrEP participants, and by underrepresentation of females in the FGDs. In addition, our findings may not reflect the views of non-English speaking individuals, those in different age groups, and those who are new to ART or PrEP. This study relied on hypothetical insights and perspectives from clients and providers on a POC-TFV test that was not clearly defined, which may have influenced participants’ opinions and may not accurately reflect their perceptions of a POC-TFV test. It is also important to note that PrEP participants in this study may have falsely perceived TFV test results as a guarantee of a level of protection against HIV acquisition, but more research is needed to assess the association between urine TFV concentrations and risk of HIV acquisition. Finally, due to the exploratory and hypothetical nature of this study, our findings should be interpreted with caution and should prompt additional research on clients’ and providers’ perceptions of POC-TFV testing.

To our knowledge, this exploratory qualitative study is the first to examine perspectives from PLWH and providers on TFV adherence monitoring and to assess clients’ and providers’ perceived utility of POC-TFV testing. Further research should assess utility, acceptability, feasibility, and effectiveness of POC-TFV testing among other populations in the U.S. and globally, particularly within HIV-endemic countries and populations with persistently low adherence levels. In doing so, this research should evaluate the effects of POC-TFV testing on clients’ adherence; retention in care; mental health such as fear, anxiety, depression, or loss of autonomy; and relationship with their provider. Additional research should also explore the utility and feasibility of some of the novel applications that were suggested by study participants, such as home-based adherence monitoring; TFV-level testing in STD clinics, clinical pharmacies, or hospital inpatient departments; or POC-TFV testing among clients with renal failure or clients with hepatitis B.

Results from this study will help guide future developments in POC-TFV monitoring tools by offering perspectives from clients and providers on preferred features and attributes of a POC-TFV test. Our findings indicate that clients and providers may benefit from a non-urine-based POC-TFV test that is low-cost and provides a long-term limit of detection. This study also contributes to the breadth of knowledge on objective antiretroviral adherence monitoring by offering important insights on the utility of POC-TFV testing and potential target populations and settings for its implementation. Our findings convey a potential need for POC-TFV adherence monitoring in clinical settings and possibly even for self-administered, home-based testing among PLWH receiving ART and people receiving PrEP. Providers may find POC-TFV testing to be particularly useful for monitoring oral PrEP adherence and for assessing discrepancies in VL results and self-reported ART adherence among PLWH.

## Data Availability

The data generated for this study are not publicly available but are available from the corresponding author on reasonable request.

## Abbreviations

ART: Antiretroviral therapy
CFAR: University of Washington Center for AIDS Research
FGD: Focus group discussion
IDI: In-depth interview
LFA: Lateral flow assay
PLWH: People living with HIV
POC: Point-of-care
PrEP: Pre-exposure prophylaxis
STD: Sexually transmitted disease
TFV: Tenofovir
VL: Viral load
